# Supplementary material for: Evaluating the association between COVID-19 and psychiatric presentations, suicidal ideation in an emergency department
Source: PLoS One. 2021 Jun 30;16(6):e0253805. doi: 10.1371/journal.pone.0253805 (PMC8244888; doi:10.1371/journal.pone.0253805)
Supplement: S1 Material — (DOCX) [file pone.0253805.s001.docx]

**S1 Material**

We have specified our general CITS regression as:

$$y_{it}=\beta_{0}+\beta_{1}COVID_{i}+\beta_{2}TREND_{t}+\beta_{3}COVID_{i}*TREND_{t}+\sum_{k>t_{0}}^{T} \beta_{k}COVID_{i}+\gamma\boldsymbol{X}_{it}$$

where *y* is an indicator for whether person *i* at time *t* presents with one of the four psychiatric conditions studied,

$\beta_{0}$ is the intercept of the comparator time series in the pre-period,

$\beta_{1}$ is the intercept for the COVID-19 time series in the pre-period,

$\beta_{2}$ is the slope of comparator time series,

$\beta_{3}$ is the slope of the COVID-19 time series,

$\beta_{k}$is the differential increase for the COVID-19 series relative to the comparator series at time, t, in the post-period (note that $t_{0}$ represents the time point of the exposure and T represents the last time point in the post-period),

and $\boldsymbol{\gamma}$ is a vector of demographic and clinical covariates.
