# Supplementary material for: Evaluating the association between COVID-19 and psychiatric presentations, suicidal ideation in an emergency department
Source: PLoS One. 2021 Jun 30;16(6):e0253805. doi: 10.1371/journal.pone.0253805 (PMC8244888; doi:10.1371/journal.pone.0253805)
Supplement: S1 Table — (DOCX) [file pone.0253805.s002.docx]

**S1 Table.** Pre-period Unadjusted Comparison of Racial/Ethnic Groups by Psychiatric Presentation

| **Racial/Ethnic Groups,**  **mean (%)** | **2018-2019 Cohort**  **(n=489)** | **2019-2020 Cohort**  **(n=467)** | ***P* Value** |
| --- | --- | --- | --- |
| White | | | |
| Suicidal Ideation | 60.3 | 57.5 | 0.45 |
| Substance Use Disorder | 42.0 | 39.7 | 0.53 |
| Affective Disorder | 34.2 | 39.4 | 0.16 |
| Psychotic Disorder | 15.4 | 10.3 | 0.04 |
| Black | | | |
| Suicidal Ideation | 52.7 | 57.0 | 0.63 |
| Substance Use Disorder | 36.4 | 36.7 | 0.97 |
| Affective Disorder | 34.5 | 41.8 | 0.40 |
| Psychotic Disorder | 25.5 | 19.0 | 0.38 |
| Asian | | | |
| Suicidal Ideation | 55.6 | 52.9 | 0.90 |
| Substance Use Disorder | 44.4 | 29.4 | 0.48 |
| Affective Disorder | 11.1 | 47.1 | 0.04 |
| Psychotic Disorder | 33.3 | 17.6 | 0.43 |
| Hispanic or Latino/a/x | | | |
| Suicidal Ideation | 66.0 | 58.5 | 0.45 |
| Substance Use Disorder | 51.1 | 41.5 | 0.34 |
| Affective Disorder | 42.6 | 28.3 | 0.14 |
| Psychotic Disorder | 14.9 | 17.0 | 0.78 |
