# Supplementary material for: Evaluating the association between COVID-19 and psychiatric presentations, suicidal ideation in an emergency department
Source: PLoS One. 2021 Jun 30;16(6):e0253805. doi: 10.1371/journal.pone.0253805 (PMC8244888; doi:10.1371/journal.pone.0253805)
Supplement: S2 Table — (DOCX) [file pone.0253805.s003.docx]

**S2 Table**. Full Regression Output for Differential Changes in Emergency Department Psychiatric Presentations in the 2019 versus 2020 Post-Periods

|  | **Psychiatric Presentation** | | | |
| --- | --- | --- | --- | --- |
|  | Suicidal Ideation *Pct point change (95% CI)* | Affective disorder *Pct point change (95% CI)* | Psychotic disorder  *Pct point change (95% CI)* | Substance use disorder  *Pct point change (95% CI)* |
| **Week since MA COVID-19 Outbreak** |  |  |  |  |
| *Differential change in the post-period^†^* |  |  |  |  |
| 1 | 6.8  (-14.2, 31.2) | -2.0 (-26.5, 22.5) | 7.1 (-11,3, 25.5) | 2.5 (-22.7, 27.8) |
| 2 | -4.1 (-29.8, 21.5) | 0.04 (-27.3, 27.4) | 4.8 (-15.7, 25.4) | 23.7* (-4.4, 51.9) |
| 3 | 21.0  (-5.3, 47.4) | 6.1 (-21.8, 34.1) | -1.0 (-22.0, 20.0) | 32.8** (4.0, 61.6) |
| 4 | -1.0 (-30.6, 28.6) | -30.8* (-62.8, 1.2) | 9.3 (-14.8, 33.3) | 25.1  (-7.8, 58.1) |
| 5 | 1.1 (-28.2, 30.4) | 3.6 (-27.7, 34.9) | 7.5 (-16.0, 31.0) | 14.4 (-17.8, 46.6) |
| 6 | 36.4** (5.3, 67.6) | 18.6 (-14.6, 51.7) | -8.5 (-33.4, 16.4) | 4.0 (-30.1, 38.2) |
| 7 | 8.8 (-23.7, 41.4) | -6.4 (-41.1, 28.3) | -4.3 (-30.4, 21.7) | 31.9* (-3.8, 67.6) |
| 8 | -7.3 (-41.2, 26.5) | -12.5 (-48.6, 23.6) | 8.0 (-19.1, 35.1) | 24.4 (-12.7, 61.5) |
| 9 | -34.9* (-69.5, -0.3) | -19.9 (-56.8, 17.0) | 8.5 (-19.2, 36.2 | 21.5 (-16.4, 59.5) |
| 10 | 0.3 (-37.1, 37.7) | -10.7 (-50.9, 29.5) | -13.3 (-43.5, 16.9) | 35.7 (-5.7, 77.0) |
| *Comparison level, post-period* |  |  |  |  |
| 1 | 84.2 (-28.4, 196.7) | -123.9** (-241.4, -6.4) | 29.0  (-60.3, 118.2) | 9.8 (-112.4, 132.0) |
| 2 | 95.6 (-16.9, 208.1) | -124.8** (-242.3, -7.4) | 25.1 (-64.1, 114.3) | -7.0 (-129.1, 115.1) |
| 3 | 89.3 (-22.7, 201.2) | -123.7** (-240.6, -6.9) | 36.1 (-52.7, 124.8) | 4.5 (-126.0, 117.0) |
| 4 | 86.1* (-11.5, 182.6) | -79.6 (-180.9, 21.7) | 8.9 (-68.1, 86.0) | 11.0 (-94.4, 116.5) |
| 5 | 81.4 (-15.6, 178.4) | -65.9 (-167.2, 35.4) | 2.0 (-75.2, 79.1) | 12.4 (-93.2, 118.0) |
| 6 | 57.6 (-39.4, 154.6) | -86.6 (-187.9, 24.3) | 18.2 (-58.8, 95.2) | 14.2 (-91.3, 119.7) |
| 7 | 68.0 (-29.0, 165.1) | -65.3 (-166.6, 36.1) | 12.2 (-64.9, 89.3) | 8.0 (-97.6, 113.6) |
| 8 | 58.1 (-37.2, 153.5) | -85.3 (-184.9, 14.3) | 4.4  (-71.3, 80.2) | 20.7 (-83.0, 124.5) |
| 9 | 89.2* (-6.2, 184.5) | -64.8 (-164.4, 34.9) | 3.4 (-72.3, 79.2) | 4.2 (-99.5, 107.9) |
| 10 | 66.2 (-29.4, 161.9) | -58.9 (-158.9, 41.1) | 17.9 (-58.1, 93.9) | 3.4 (-100.6, 107.5) |
| **Comparison level, pre-period** | 46.4*** (29.9, 62.9) | 64.4*** (48.0, 80.8) | 12.4** (-0.2, 25.1) | 43.9*** (26.7, 61.2) |
| **Comparison trend, pre-period** | -0.4 (-3.2, 2.5) | -5.4*** (-8.5, -2.2) | 0.2 (-2.0, 2.5) | -1.1 (-4.2, 2.1) |
| **Treated level, pre-period** | -4.3 (-17.6, 9.0) | -5.2 (-8.5, 19.3) | -8.9 (-19.5, 1.7) | 5.1 (-9.5, 19.6) |
| **Treated trend, pre-period** | 0.2 (-2.0, 2.4) | -0.03 (-2.4, 2.0) | 0.6 (-1.2, 2.3) | -1.2 (-3.6, 1.2) |
| **Sex** | 3.8*  (-0.4, 8.1) | -0.1 (-8.2, 2.2) | -1.5 (-5.0, 1.9) | -2.3 (-7.0, 2.3) |
| **Any SUD** | 24.6*** (19.9, 29.4) | -- | -- | -- |
| **Any affective disorder** | 13.0*** (8.0, 18.0) | -- | -- | -- |
| **Any psychotic disorder** | -23.3*** (-29.8, -16.8) | -- | -- | -- |
| ***Race/Ethnicity*** |  |  |  |  |
| *White* | REF | REF | REF | REF |
| *Black* | -0.6 (-6.9, 5.4) | 1.9 (-4.4, 8.2) | 10.8 (0.9, 20.6) | -9.0 (-22.5, 4.5) |
| *Asian* | 2.1 (-26.6, 23.2) | -3.5 (-15.8, 8.8) | 15.3** (-4.3, 35.0) | 4.8 (-22.0, 31.7) |
| *American Indian/Alaskan Native* | -29.1* (-60.9, 2.6) | 24.7 (-8.5, 57.9) | 0.7 (-12.1, 13.6) | 1.7 (-15.9, 19.3) |
| *Other* | 13.0** (2.2, 23.9) | 1.5 (-9.8, 12.8) | 3.6 (-3.8, 11.1) | 2.7 (-7.4, 12.9) |
| *Missing* | 10.5 (-17.9, 38.9) | -3.4 (-33.1, 26.3) | -7.0 (-25.2,15.4) | -8.0 (-36.1, 20.0) |
| **Hispanic/Latino** | -7.9* (-17.3, 15.0) | -1.4 (-11.3, 8.4) | -5.0 (-2.7, 11.5) | 0.5 (-9.3, 10.4) |
| ***Payer*** |  |  |  |  |
| *MA Medicaid* | REF | REF | REF | REF |
| *Private Ins.* | -0.4 (-5.8, 4.9) | -2.3 (-7.8, 3.3) | 3.2 (-1.1, 7.5) | -3.1 (-9.0, 2.8) |
| *Medicare* | 2.2 (-3.9, 8.2) | -3.0 (-9.3, 3.2) | 5.2** (0.5, 10.0) | -4.0 (10.6, 2.5) |
| *Uninsured* | -1.4 (-11.8, 9.1) | -2.4 (-13.2, 8.5) | -1.3 (-9.7, 7.0) | 11.6** (0.3, 23.1) |
| *Other public payer* | 1.0 (-7.0, 9.0) | -3.0 (-11.3, 5.4) | 2.6 (-3.9, 9.1) | -7.8 (-16.7, 1.2) |
| **Age** | 0.1 (-0.05, 0.3) | 0.00 (-0.2, 0.2) | -0.03 (-0.2, 0.1) | 0.01 (-0.2, 0.2) |
| **Day fixed effect** |  |  |  |  |
| *Monday* | REF | REF | REF | REF |
| *Wednesday* | -2.9 (-8.2, 2.3) | -2.5 (-7.9, 3.0) | 2.1 (-2.0, 6.3) | -1.8 (-7.5 4.0) |
| *Friday* | -9.3*** (-14.6, -4.1) | -6.8 (-12.3, -1.3) | 2.1 (-2.1, 6.3) | 1.0 (-4.8, 6.8) |
| **Month fixed effect** |  |  |  |  |
| *January* | REF | REF | REF | REF |
| *February* | 2.8 (-9.4, 15.0) | 16.5** (3.8, 29.3) | -0.3 (-10.0, 9.5) | 10.5 (-2.9, 23.9) |
| *March* | -91.5 (-207.7, 24.7) | 158.1** (36.9, 279.3) | -35.3 (127.4, 56.8) | 10.4 (-115.7, 136.6) |
| *April* | -69.1 (-171.5, 33.3) | 138.4** (31.7, 245.2) | -15.0 (-96.3, 66.3) | -1.8 (-113.1, 109.5) |
| *May* | -64.4 (-165.1, 36.3) | 144.0** (39.1, 249.0) | -10.3 (-90.2, 69.6) | -0.2 (-109.6, 109.2) |
| *December* | 2.6 (-9.8, 15.0) | -22.9*** (-35.9, -10.0) | 0.03 (-9.9, 10.0) | -4.2 (-17.8, 9.4) |
| **Total ED visits** | 7.0*** (4.5, 9.4) | -6.8*** (-9.3, 27.0) | 3.0** (1.0, 4.9) | 6.0*** (3.3, 8.6) |
| **R^2^** | 0.18 | 0.04 | 0.04 | 0.01 |
| **N** | 1867 | 1867 | 1867 | 1867 |
| **Model F-test** test statistic; fp-value; degrees of freedom | 8.72***  ; <0.001;  46, 1830 | 2.56***; <0.001; 43, 1833 | 1.23; 0.15;  43, 1833 | 1.67***; 0.004;  43, 1833 |

SOURCES/NOTES:

**Source** Authors’ analysis of EHR data from psychiatric consult visits

**Notes** Estimates are from a general CITS regression analysis with a treatment effect for each post-period week, using a linear probability model. All models were adjusted with patient age, sex, racial classification, ethnicity, month and day-of-week fixed effects, payer, and the total number of psychiatric consults. The suicidal ideation regression was also adjusted with whether a patient also had an affective disorder, psychotic disorder, and substance use.

^†^This is the difference between the change in proportion of ED visits with a psychiatric presentation from the pre-period to post-period for the COVID-19 series and the change in the proportion of ED visits with a psychiatric presentation from the pre-period to the post-period in the comparison series (quantity of interest)**.**

*p<0.1, **p<0.05, ***p<0.01)
