# Supplementary material for: Evaluating the association between COVID-19 and psychiatric presentations, suicidal ideation in an emergency department
Source: PLoS One. 2021 Jun 30;16(6):e0253805. doi: 10.1371/journal.pone.0253805 (PMC8244888; doi:10.1371/journal.pone.0253805)
Supplement: S3 Table — (DOCX) [file pone.0253805.s004.docx]

**S3 Table.** Differential change in suicidal ideation presentations between COVID-19 time series (Dec 2019 – May 2020) and comparator time series (Dec 2018 – May 2019) from pre-period (Dec – Feb) to post-period (Mar – May) among all ED psychiatric presentations, varying adjustment for other co-occurring psychiatric conditions

|  | **Adjustment for co-occurring conditions** | | | | |
| --- | --- | --- | --- | --- | --- |
|  | None *Pct point change (95% CI)* | Affective disorder *Pct point change (95% CI)* | Psychotic disorder  *Pct point change (95% CI)* | SUD  *Pct point change (95% CI)* | All 3 conditions Pct point change  (95% CI) |
| **Differential change in the week since MA COVID-19 Outbreak***^†^* |  |  |  |  |  |
| 1 | 5.6 (-18.9, 30.0) | 5.3 (-19.1, 29.6) | 7.4 (-16.2, 31.1) | 6.0 (-17.8, 29.7) | 6.8  (-14.2, 31.2) |
| 2 | 1.1 (-26.2, 28.3) | 1.2 (-25.9, 28.32) | 2.4 (-23.9, 28.7) | -5.1 (-31.5, 21.4) | -4.1 (-29.8, 21.5) |
| 3 | 30.2** (2.2, 58.2) | 29.5** (1.6, 57.3) | 29.3** (2.2, 56.3) | 22.6 (-4.6, 49.8) | 21.0  (-5.3, 47.4) |
| 4 | 0.2 (-31.3, 31.6) | 3.4 (-27.9, 34.7) | 2.4 (-28.0, 32.8) | -6.4  (-36.9, 24.1) | -1.0 (-30.6, 28.6) |
| 5 | 2.3 (-28.9, 33.5) | 1.9 (-29.1, 33.0) | 4.4 (-25.8, 34.5) | 0.3 (-29.9, 30.6) | 1.1 (-28.2, 30.4) |
| 6 | 41.4*** (8.3, 74.5) | 39.0** (6.0, 71.9) | 38.0** (6.1, 70.0) | 41.7** (9.6, 73.8) | 36.4** (5.3, 67.6) |
| 7 | 17.0 (-17.6, 51.6) | 17.5 (-17.0, 51.9) | 15.1 (-30.4, 21.7) | 9.5 (-24.1, 43.1) | 8.8 (-23.7, 41.4) |
| 8 | -4.2 (-40.2, 31.9) | -3.3 (-39.1, 32.6) | -2.1 (-36.9, 32.7) | -9.8 (-44.7, 25.2) | -7.3 (-41.2, 26.5) |
| 9 | -34.6* (-71.4, 2.2) | -32.8* (-69.5, 3.8) | -32.1* (-67.7, 3.4) | -38.6** (-74.3, -2.9) | -34.9* (-69.5, -0.3) |
| 10 | -0.3 (-2.6, 2.1) | 10.2 (-29.4, 49.8) | 4.6 (-33.9, 43.0) | 2.7 (-35.9, 41.3) | 0.3 (-37.1, 37.7) |
| **R^2^** | 0.07 | 0.06 | 0.13 | 0.13 | 0.18 |
| **N** | 1867 | 1867 | 1867 | 1867 | 1867 |
| **Model F-test** test statistic; p-value; degrees of freedom | 3.15***; <0.001; 44, 1833 | 3.51***; <0.001; 44,1832 | 6.36***; <0.001; 44, 1832 | 5.98***; <0.001; 44, 1832 | 8.72***; <0.001;  46, 1830 |

SOURCES/NOTES:

**Source** Authors’ analysis of EHR data from psychiatric consult visits

**Notes** Estimates are from a general CITS regression analysis with a treatment effect for each post-period week, using a linear probability model. All models were adjusted with patient age, sex, racial classification, ethnicity, month and day-of-week fixed effects, payer, and the total number of psychiatric consults. The exposure for both time series was the two-week period of late February to mid-March. In 2020, this time period represents the time where COVID-19 cases were growing in Boston, MA and the city/state governments were implementing interventions to curb the spread of the disease. We varied adjusting for other co-morbid psychiatric conditions in each regression above. *^†^*This is the difference between the change in proportion of ED visits with a psychiatric presentation from the pre-period to post-period for the COVID-19 series and the change in the proportion of ED visits with a psychiatric presentation from the pre-period to the post-period in the comparison series (quantity of interest).

* p<0.10; **p<0.05; ***p<0.01
