# Supplementary material for: Evaluating the association between COVID-19 and psychiatric presentations, suicidal ideation in an emergency department
Source: PLoS One. 2021 Jun 30;16(6):e0253805. doi: 10.1371/journal.pone.0253805 (PMC8244888; doi:10.1371/journal.pone.0253805)
Supplement: Attachment — Submitted filename: Response to Reviewers.docx [file pone.0253805.s006.docx]

**Reviewer #1**: I think the authors have done a commendable job with the revision— the clarifications and additional information were very helpful. This paper will be a nice contribution to the literature in this area.

**Many thanks for your generous comments on our manuscript! We believe they have significantly improved the quality of the work we submit now.**

I have only a few remaining minor comments for the investigators’ consideration. The page numbers refer to the tracked version rather than the clean version:

1. As previously noted, the study aims section (p. 4, last paragraph of the Introduction) is a bit vague and it seems that outcomes are only clarified for the reader 5 pages later (p. 10). It might be useful to change “psychiatric complaints” to “certain psychiatric complaints, and then to note in parentheses “(a number of selected substance use, mood, and psychotic disorders or suicidal ideation)” or similar phrasing-- in other words, provide a clearer sense of the study endpoints. Just a thought.

**We agree that our aims in the last paragraph of the introduction could use clarification. We appreciate your suggestions and have included the word “certain” to qualify “psychiatric disorders.” We also now indicate in parentheses the outcomes of interest: “We used the period following the initial outbreak of COVID-19 in Massachusetts to assess the association between the pandemic and patients presenting with certain psychiatric complaints (a number of selected mood, psychotic, and substance use disorders, as well as suicidal ideation) to a tertiary care hospital Emergency Department (ED).”**

2. The statistical analysis section (top of p. 7) indicates that “a general rather than a linear CITS” was used to reduce assumptions, but the subsequent newly added sentence states that “A general CITS assumes that any differential growth… is linear.” For readers unschooled in CITS, like myself, that might seem at first blush like a logical contradiction, and perhaps the authors might take another look at those 2 sentences for clarity. Additionally, the new text on the bottom of the page states that “patients were allowed to appear in more than one of these periods,” and it might be helpful to briefly assure readers that this does not introduce problematic dependency in the data.

**Thank you for highlighting these points, which we agree would all benefit from clarification.**

**A linear CITS assumes that the evolution of the outcome (i.e., the probability of a patient showing up to the ED with a psychiatric presentation) is linear over the course of the study, which makes a parametric assumption about the post-period outcomes (i.e., that it follows the form of a line). However, a general CITS does not make an assumption about the post-period outcomes follow any shape (liner, quadratic, etc.). In a general CITS, the outcome is not assumed to be of a specific functional form. Instead, general CITS makes the assumption that the difference between the two groups is evolving linearly.**

**To clarify this point to our readers, we have included the following changes in the manuscript text in the second full paragraph of the Methods section, Study design sub-section: “Finally, we use a general CITS, rather than a linear CITS, to minimize the parametric assumptions made regarding the outcome’s evolution. A linear CITS assumes that the outcome is evolving linearly in the pre- and post-period (24). On the other hand, a general CITS assumes that any difference between the two groups is evolving linearly but does not make any assumption about the outcome’s parametric form (i.e., does not assume a linear, quadratic, or other form). We conducted an event study to visually assess the plausibility of these assumptions and found differential trends (i.e., non-parallel) in the pre-period suggesting that a standard difference in difference approach would not be appropriate.”**

**Regarding the issue of multiple presentations, we treat each of the four periods (2018-2019 pre, 2018-2019 post, 2019-2020 pre, 2019-2020 post) as a cohort in this cross-sectional study. We do make adjustments for patients presenting multiple times in one of the study periods, which we now specify in the antepenultimate paragraph in the Methods section, Study design sub-section: “We adjusted the models for the total number of emergency department visits per patient per study period.”**

3. In the Results section regarding findings for the post-exposure periods, I think it would be important to indicate more explicitly that separate analyses were run for each of 10 weeks, if I understand correctly. That is, the text describing the significant results for SI at six weeks (p. 15, line 282), and the passage describing significant results for SUD at three weeks (p. 16, line 302) should note more clearly that each of these findings represent one of 10 analyses for each outcome, if that is accurate. (This approach is mentioned in the Data Analysis section of the Methods but should be specified in the Results.)

**Thank you for this helpful suggestion. We agree that this point certainly needs clarification.**

**We did not run a separate analysis for each of the 10 weeks in the post-period. Rather, our regression specification includes an indicator variable for each post-period week. We include the indicator variable and an interaction between the treatment variable and the weekly indicator variable. Thus, each of these interactions represent the differential change for this week between the COVID-19 series and comparator series from the average pre-period level. *We include our single regression formula at the beginning of the manuscript’s Supplemental Material to provide further explanation of the regression analysis conducted. In addition, we removed the “s” from “general CITS regression(s)” at the beginning of our Supplemental materials section to clarify that the analyses presented derive from a single regression specification.***

**We have added a sentence in the last paragraph of the Methods section, Data analysis sub-section to further clarify this point, as well: “*In other words, our regression specification included an indicator variable for each post-period week. We and interacted the indicator variable with the treatment variable, and each of these interactions represent the differential change for the week between the COVID-19 series and comparator series as compared to the average pre-period levels.”***

4. On p. 15 in the Results section, there is ambiguity regarding the type of analysis described in the 3rd sentence on the page (lines 281-285), since the 1st sentence refers to adjusted model and the 2nd sentence refers to the unadjusted analyses. It may be helpful to clarify for readers that the 3rd sentence is once again referring to the adjusted analyses. In addition, should the text mention that there was an unexpected marginally significant decrease in SI at week 9? This finding does not seem to be mentioned in the Results section, and instead emerges for the first time in the Discussion (p. 18, lines 235-360). Was there a significant decrease in SI for those presenting with psychotic disorders, and was there a significant increase in SI for those from “other” racial/ethnic backgrounds relative to whites (as seems to be suggested in Appendix 2)?

***We agree that the first sentences in the Results section paragraph following Fig 3 were confusing to follow. We have improved clarity by reordering the sentences such that we mention the unadjusted models first, and then discuss the adjusted models following.***

***Thank you for the suggestion that we name the non-significant decrease in SI at week 9 in the Results section. We now do this at the end of the Results section paragraph following Fig 3: “Additionally, at nine weeks after the exposure (May 4-9, 2020), there was a differential decrease in the proportion of psychiatric presentations to the ED with suicidal ideation (34.9 percentage points; 95% CI; -69.5, -0.3), though this change was not statistically significant (p = 0.06).”***

***Your questions regarding Appendix Table 2 are helpful. In the “Any SUD,” “Any affective disorder,” “Any psychotic disorder” and “Other” race/ethnic background rows, you are correct that the confidence intervals do not cross 0 for the suicidal ideation column. This indicates that, over the course of the study, after accounting for all other covariates, including the exposure (COVID-19 outbreak), there is a relationship between suicidal ideation and other psychiatric conditions and/or demographic characteristics. In other words, the probability of presentation with suicidal ideation increases with the presence of co-occurring SUD, increases with co-occurring affective disorders, decreases with co-occurring psychotic disorders, and increases in people with “other” race/ethnic backgrounds. These changes do not have do to with COVID.***

***We are happy to provide additional text clarifications on Appendix Table 2 at the Editor and Reviewer’s discretion.***

5. The new text in the Results section includes a helpful summary of changes in SI based on unadjusted analyses (p. 16). Should the text also mention that there was a significant increase in SI at week 6 in the unadjusted model (as suggested in Appendix 3)?

**Thank you for highlighting this point, which could certainly use clarification. We now include more explanation regarding Appendix Table 3 in the paragraph following Fig 3 in the Results section: “*We present data in Appendix Table 3 for suicidal ideation, sequentially removing and re-adding other certain psychiatric presentations to the model. Our results change modestly as a result of this different regression specification. Specifically, there is a significant increase in presentations with suicidal ideation at weeks three and six. However, once adjustment for SUD is included in the model at week three, the differential change is no longer significant because this increase was mediated by the co-occurring SUD diagnoses. However, even after the adjustments for co-occurring psychiatric conditions are added at week six, the differential change persists.”***

6. In Appendix Table 2 and 3, it is not clear whether the model as a whole was significant for each outcome (i.e., the sample size and R square is presented for each outcome in Table 2, which is great, but not the significance level for the overall model for each outcome). More generally, I had a bit of difficulty interpreting this table, no doubt due to my limited familiarity with CITS, but the authors might wish to explain this Appendix more clearly (e.g., how does comparison level differ from differential level?).

**We agree that Appendix Tables 2 and 3 may be challenging to follow.**

**First, we now include the global F-test statistic, p-value, and degrees of freedom to Appendix Table 2 and 3. In Appendix Table 2, the F-test statistics demonstrate that for suicidal ideation, affective disorders, and SUD, the models are statistically significant. For psychotic disorders, the overall model is not statistically significant, suggesting that this model does not explain the variation in the outcome better than the intercept-only model, suggesting that changes in the presence of psychotic disorder may not be attributable to COVID-19 and may be more complex than the current regression models can explain.**

**Second, we have re-arranged Appendix Table 2 to first display the quantities of interest (i.e., the treatment effects).**

**Third, we have added text in the notes of both Tables to help readers understand what these estimates represent: “This is the difference between the change in proportion of ED visits with a psychiatric presentation from the pre-period to post-period for the COVID-19 series and the change in the proportion of ED visits with a psychiatric presentation from the pre-period to the post-period in the comparison series (quantity of interest).”**

7. The Discussion section indicates that there was a differential decrease in SI at nine weeks after the exposure (p. 18, lines 357-360). It might be useful to clarify that the text is referring to the adjusted analyses.

**We agree that this clarification would prove helpful. We now include the qualifier “in the adjusted analyses,” in this sentence.**

Other MINOR comments:

1. It might be helpful to specify the CITS abbreviation the first time the term is used in the paper (e.g., insert “CITS” in parentheses after “Comparative interrupted time series” on p. 6 line 100).

**Thank you for this catch! We now include the abbreviation after the first time the term is used in the paper.**

2. The change in terminology from “intervention” to “exposure” is appreciated. To avoid confusion, the authors might wish to make the same change in the remainder of the manuscript (i.e., p. 6, lines 110-112; p. 8, line 150; p. 11, lines 207 and 225; p. 15, line 293; p. 22, lines 439-441; Appendix Table 3) though they

**Thank you for this helpful suggestion – we have changed the terms from “intervention” to “exposure” throughout the manuscript.**

3. On p. 6, line 107, it may be helpful to change “psychiatric presentation” to “selected psychiatric presentations” or “certain psychiatric presentations” or comparable language. On p. 11, the authors might wish to change the phrasing in the first and last paragraphs to the past tense.

**Thank you, we agree these clarifications would be useful for readers. We have amended to include “certain.” We have also amended the two indicated paragraphs to read in the past tense.**

4. On p. 12, for clarity, it would be useful to reiterate the time intervals for readers, e.g., by inserting in parentheses “(Dec. 2019- Feb. 2020)” after “COVID-19 pre-period” and by inserting “(Dec. 2018- Feb. 2019)” after “comparator pre-period.”

**Thank you for this helpful suggestion. We now include the following clarifications: In exploring pre-period differences, we found that the COVID-19 pre-period (December 23, 2019 – February 24, 2020) cohort had a higher proportion of psychiatric ED consultations involving people from a minority racial group than did the comparator pre-period (December 24, 2018 – February 25, 2019) (Table 1).”**

5. On p. 18, line 355, to facilitate interpretation it may be helpful to note the location of the study cited in this sentence (e.g., perhaps insert “in the US” after “cross-sectional study”).

**We agree that this would be a helpful clarification. The sentence now reads: “This finding regarding increased pandemic-related suicidal ideation, as well as our finding regarding increased presentations with SUD, is corroborated by another recent large-scale cross-sectional study in the United States (32).”**

6. On p. 20, it may be helpful to clarify the direction of the effects (perhaps by changing “White” to “fewer White,” and changing “Asian” to “more Asian” on line 397, and by inserting “in the 2019-2020 cohort” after “with affective disorders” on line 398).

**We certainly agree that this clarification would be helpful to readers. We have made the suggested edits and the sentence now reads: “When we further explore this difference by separating the groups by psychiatric presentations, statistically significant differences are isolated to fewer White patients presenting with psychotic disorders and more Asian patients presenting with affective disorders in the 2019-2020 cohort.”**

7. Appendix Table 3, it may be useful to specify the demographic/temporal covariates that were included in all these analyses, in a note at the bottom of the table, to avoid a misimpression. In the title, after “Differential change in suicidal ideation presentations,” the authors might consider inserting “from ____ to ____” (i.e., specify the comparison conditions).

**Thank you for this helpful suggestion. We have amended the title of the table to better represent what we are showing in the table and have added notes to the table to further explain our covariate adjustment and the meaning of the coefficients in the table.**

Good luck with your work!

**Many thanks for your patience and generosity in reviewing and offering suggestions to our paper.**
